# The rapid development of computational toxicology

**DOI:** 10.1007/s00204-020-02768-5

**Published:** 2020-05-07

**Authors:** Hermann M. Bolt, Jan G. Hengstler

**Affiliations:** Department of Toxicology, Leibniz Research Centre for Working Environment and Human Factors at TU Dortmund (IfADo), Ardeystr. 67, 44139 Dortmund, Germany

Ten years ago, Archives of Toxicology has issued an Editorial by R.D. Combes (2010) entitled “Is computational toxicology withering on the vine?” At that time, there was an ongoing development in the computational handling of molecular descriptors (Kirkovsky et al. 1998; Dorn et al. 2008) and in statistical methodologies (Valerio et al. 2010). There was a perspective for applications of computational methods in general both in pharmacology (Valerio 2009) and in regulatory toxicology (Lilienblum et al. 2008). However, a weakness hindering wider application of statistical in silico systems was often missing or weak external validation, mostly due to a lack of a sufficient number of test chemicals that were not used in the training set (Combes 2010). Explicit guidance on how to use the output of computational models in the range of regulatory context had not yet been developed (Mostrag-Szlichtyng et al. 2010).

In the following, important research lines of computational toxicology were the development and refinement of computational models for relevant toxicological endpoints, such as liver injury, cardiotoxicity, renal toxicity and genotoxicity (Ekins 2014). Best predictions were obtained combining different tools, to comply with particular situations (Carrió et al. 2016).

As far as regulatory acceptance is concerned, a decisive breakthrough was the integration of (Q)SAR methodologies into the guideline ICH M7 “Assessment and control of DNA-reactive (mutagenic) impurities in pharmaceuticals to limit potential carcinogenic risk”, issued by the International Council for Harmonisation of Technical Requirements for Pharmaceuticals for Human use (Amberg et al. 2016; Hasselgren and Myatt 2018): in the absence of adequate experimental data, results of two complementary (Q)SAR methodologies (rule based and statistically learning based) were considered adequate to support an initial hazard classification (Tung et al. 2020), which may be followed by an assessment of additional information in an expert review to support or refute the computational predictions (Amberg et al. 2016). Similarly, also in regulatory fields other than pharmaceuticals, in silico models received increased acceptance. An example is prioritization of heat-induced food contaminants for mutagenicity and carcinogenicity testing (Frenzel et al. 2017).

A burst in manuscript submissions to “Archives of Toxicology” covering the in silico/computational field is noticed since 2019, signaling both increased scientific importance and regulatory relevance in the twenty-first century of this research area (Krewski et al. 2020). The following trends are visible:

- Advanced computational methodologies (Kusko and Hong 2019) enable progress in fields that had been difficult to cover before. Apart from the more classical applications, substantial progress is now noted in areas such as exposure assessment (Krewski et al. 2020), sensitization (Tung et al. 2019), neurotoxicity (Kosnik et al. 2020) and even developmental/reproductive toxicity (Manganelli et al. 2020; Tung et al. 2020).
- Perspectives are emerging for computational approaches to predict the toxicity of nanomaterials (Buglak 2019) and of chemical mixtures (Klar and Leszczynski 2019).
- Computational toxicology continues to assist in refining PBPK modelling (Savvateeva et al. 2020) and in exploring modes of toxic action (Ning et al. 2019; Yang et al. 2019; Hengstler et al. 2020).

A current tendency, both in the United States (Kosnik et al. 2020) and in Europe (Mahony et al. 2018), is the availability of curated public and commercial databases in the future (“Big Data”), suitable for application of hierachial clustering and machine learning. A close interplay is envisaged between such Big Data, the refinement of predictive models, toxicological experimentation and mechanistic modelling (Kleinstreuer et al. 2020).

Given this exciting development, further submissions of manuscripts from these fields to Archives of Toxicology are highly encouraged!
